# Provision and standards of care for treatment and follow-up of patients with Autoimmune Hepatitis (AIH)

**DOI:** 10.1136/flgastro-2020-101661

**Published:** 2021-05-18

**Authors:** Victoria Mary Gordon, Ratul Adhikary, Guruprasad P Aithal, Victoria Appleby, Debasish Das, James Day, Toby Delahooke, Selena Dixon, David Elphick, Claire Hardie, Michael Heneghan, Barbara Hoeroldt, Patricia Hooper, John Hutchinson, Rebecca L Jones, Faisal Khan, Jane Metcalf, Alick Nkhoma, Stavroula Pelitari, Martin Prince, Annell Prosser, Sushma Saksena, Vinay Sathyanarayana, Deven Vani, Andrew Yeoman, Dermot Gleeson

**Affiliations:** 1 Hepatology, University Hospitals Coventry and Warwickshire NHS Trust, Coventry, UK; 2 Calderdale Royal Hospital, Halifax, UK; 3 Nottingham Digestive Disease Centre, NIHR Biomedical Research Unit, Nottingham University, Nottingham, UK; 4 Gastroenterology, Bradford Teaching Hospitals Foundation Trust, Bradford, UK; 5 Kettering General Hospital, Kettering, UK; 6 Addenbrooke's Hospital, Cambridge, UK; 7 Leicester Royal Infirmary, Leicester, UK; 8 Airedale NHS Foundation Trust, Keighley, UK; 9 Chesterfield Royal Hospital, Chesterfield, UK; 10 Freeman Hospital, Newcastle upon Tyne, UK; 11 Institute of Liver Studies, King's College Hospital NHS Foundation Trust, London, UK; 12 Rotherham General Hospitals NHS Trust, Rotherham, UK; 13 Derby Teaching Hospitals NHS Foundation Trust, Derby, UK; 14 Hepatology, York Teaching Hospital NHS Foundation Trust, York, UK; 15 Department of Hepatology, St James's University Hospital, Leeds, UK; 16 Doncaster Royal Infirmary, Doncaster, UK; 17 North Tees University Hospital, North Tees, UK; 18 Gastroenterology Department, University Hospitals of North Midlands NHS Trust, Stoke-on-Trent, UK; 19 Hepatology, University Hospitals Coventry and Warwickshire NHS Trust Gastroenterology, Coventry, UK; 20 Manchester Royal Infirmary, Manchester, UK; 21 Singleton Hospital, Swansea, UK; 22 The Royal London Hospital, London, UK; 23 Barnsley District General Hospital NHS Trust, Barnsley, UK; 24 Mid Yorkshire Hospitals NHS Trust, Wakefield, UK; 25 Gwent Liver Unit, Aneurin Bevan University Health Board, Newport, UK; 26 Hepatology, Royal Hallamshire Hospital, Sheffield, UK

**Keywords:** autoimmune hepatitis, audit

## Abstract

**Background:**

Autoimmune hepatitis (AIH) is a substantial UK health burden, but there is variation in care, facilities and in opinion regarding management. We conducted an audit of service provision and care of patients with AIH in 28 UK hospitals.

**Methods:**

Centres provided information about staffing, infrastructure and patient management (measured against predefined guideline-based standards) via a web-based data collection tool.

**Results:**

Hospitals (14 university hospitals (UHs), 14 district general hospitals (DGHs)) had median (range) of 8 (3–23) gastroenterologists; including 3 (0–10) hepatologists. Eight hospitals (29%, all DGHs) had no hepatologist. In individual hospital departments, there were 50% (18–100) of all consultants managing AIH: in DGH’s 92% (20–100) vs 46% (17–100) in UHs. Specialist nurses managed AIH in only 18%. Seventeen (61%) hospitals had a histopathologist with a liver interest, these were more likely to find rosettes than those without (172/795 vs 50/368; p<0.001).

Of 999 steroid-treated patients with ≥12 months follow-up, 25% received steroids for <12 months. After 1 year of treatment, 82% of patients achieved normal serum alanine aminotransaminase (ALT); this was higher in UHs than DGHs. Three-monthly liver blood tests were inadequately recorded in 26%. Of potentially eligible patients with liver decompensation, transplantation was apparently not considered in 5% (n=7). The same standards were attained in different types of hospital.

**Conclusion:**

Management of AIH in UK hospitals is often shared between most gastroenterologists. Blood test monitoring and treatment duration are not always in line with recommendations. Some eligible patients with decompensation are not discussed with transplant teams. Care might be improved by expanding specialist input and management by fewer designated consultants.

**Summary box**

**What is already known on this topic?**

- Autoimmune hepatitis (AIH) is a substantial health burden in the UK, but there is variation in care, facilities and in opinion regarding management.^7 11 19^

**What are the new findings?**

- In a multicentre audit of service delivery and standards of care for AIH in 28 hospitals we found that:
- One-third of hospitals lacked a hepatologist, and only 18% had a specialist liver nurse managing AIH. In many hospitals, care of AIH was shared among most gastroenterologists.
- Fourty per cent of hospitals did not employ a histopathologist with a specialist liver interest; in these, histological features supporting AIH were reported less commonly.
- One quarter of patients did not continue corticosteroids for 1 year.
- Liver blood test monitoring was less frequent than current guidance^8^ recommends in 25%.
- Referral to/discussion with a transplant team was not done in 5% of patients with decompensation who were potentially eligible for transplantation.

**Summary box**

**How might it impact on clinical practice in the foreseeable future?**

- These results support the case for:
- (a)Further development of UK liver specialist services including specialist nurses and histopathologists.
- (b)Patients with AIH being managed by fewer Ggastroenterologists/hepatologists.
- (c)Referral of all eligible patients for liver transplantation.
- (d)Databases in each participating centre to improve monitoring and care.
- (e)Regional networks to discuss challenging cases.

## Introduction

Autoimmune hepatitis (AIH), although considered a rare disease, has a prevalence and incidence in Western Europe of 24/100 000 and 1.7/100 000, respectively,^1–3^ equivalent to a UK District General Hospital (DGH) serving approximately 250 000 people, having 60 patients with AIH attending it, and 4–5 new patients per year. AIH is usually a life-long disease, which even with standard treatment can result in progressive liver disease and excess mortality.^4–6^

In 2012, a nationwide survey of UK gastroenterologists revealed variation in the approach to managing patients with AIH.^7^ There are no validated standards of care for the management of AIH, but there are UK, European and American guidelines.^8–10^

Although liver disease has been highlighted as a health priority, resources remain inadequate. In 2011, a UK medical workforce census^11^ highlighted fewer hepatologists in DGHs compared with university hospitals (UHs). Of 146 responding hospitals, 71% had no hepatologist and 16% had no hepatologist nor gastroenterologist with a specialist interest in hepatology. A 2018 survey of 88 UK Trusts by the BSG Clinical Services and Standards committee found 37 of 63 DGHs (compared with 1 of 27 UHs) had no hepatologist.^12^ This deficit in consultant workforce has been emphasised in the Lancets 2020 annual report.^13^

In April 2012, a group of gastroenterologists and hepatologists from across the UK met to plan a multicentre management and outcome audit of patients with AIH. We subsequently completed an audit of diagnosis, management and outcome of AIH in 28 UK centres. We recently reported on patient characteristics at diagnosis of AIH and performance regarding preagreed diagnostic standards.^14^ Here, we report on resources and on adherence to preagreed management standards.

## Methods

We arrived at our audit standards based on the 2012 meeting and on published BSG and AASLD Guidelines.^8 10^

Twenty-eight participant centres identified patients using our capture strategy, developed and validated in Sheffield.^14^ Information on staffing, resources and service provision was collected via electronic proformas, provided by each centre Clinical Coordinator by 30 November 2015. Information on drug treatment was collected between 1 January 2014 and 30 November 2015. Anonymous patient data were entered into a data web-based data collection platform (Formic) and pooled on an encrypted N3-server in Sheffield.

Cirrhosis was determined by liver biopsy, presence of varices, ascites and/or on Fibroscan. Clinical decompensation was defined as ascites, variceal bleeding or encephalopathy.

Results, unless stated otherwise, are expressed as median (range). Z test was used to calculate proportional differences and Mann-Whitney U test used for nonparametric independent samples. SPSS and GraphPad software were used to analyse data. p<0.05 was considered statistically significant.

## Results

### Resources

Of 28 centres, 14 were DGHs; 9 with >500 beds and 14 were UHs; all with >500 beds. Sixteen hospitals accepted hepatology referrals from other hospitals.

Hospitals had a median (range) 8 (3–23) consultant gastroenterologists each. Of these, 3 (0–10) were hepatologists (liver disease >70% of workload), which were more prevalent in UHs than DGHs (table 1). There were 0 (0–4) gastroenterologists per hospital who had an interest in hepatology (gastroenterologists with an interest in hepatology (GIH)) (liver disease 40%–70% of workload), these were more prevalent in DGHs (table 1).

**Table 1:** Staffing and infrastructure

	Number of hepatologists	Number of GIH	Number of specialist nurses	Number of liver specialist nurses	Histopathologist with liver specialist interest	Clinical histopathology meeting
	Median (range)				%	%
UH (n=14)	3 (1–10)	0 (0–3)	6 (2–16)	2.5 (0–7)	86	86
DGH (n=14)	0 (0–2)	1 (0–4)	2 (0–4)	0.5 (0–3)	35	57
P value	<0.001	0.04	<0.001	0.002	0.006	0.09

DGH, district general hospital; GIH, gastroenterologists with an interest in hepatology; UH, university hospital.

**Figure 1:**
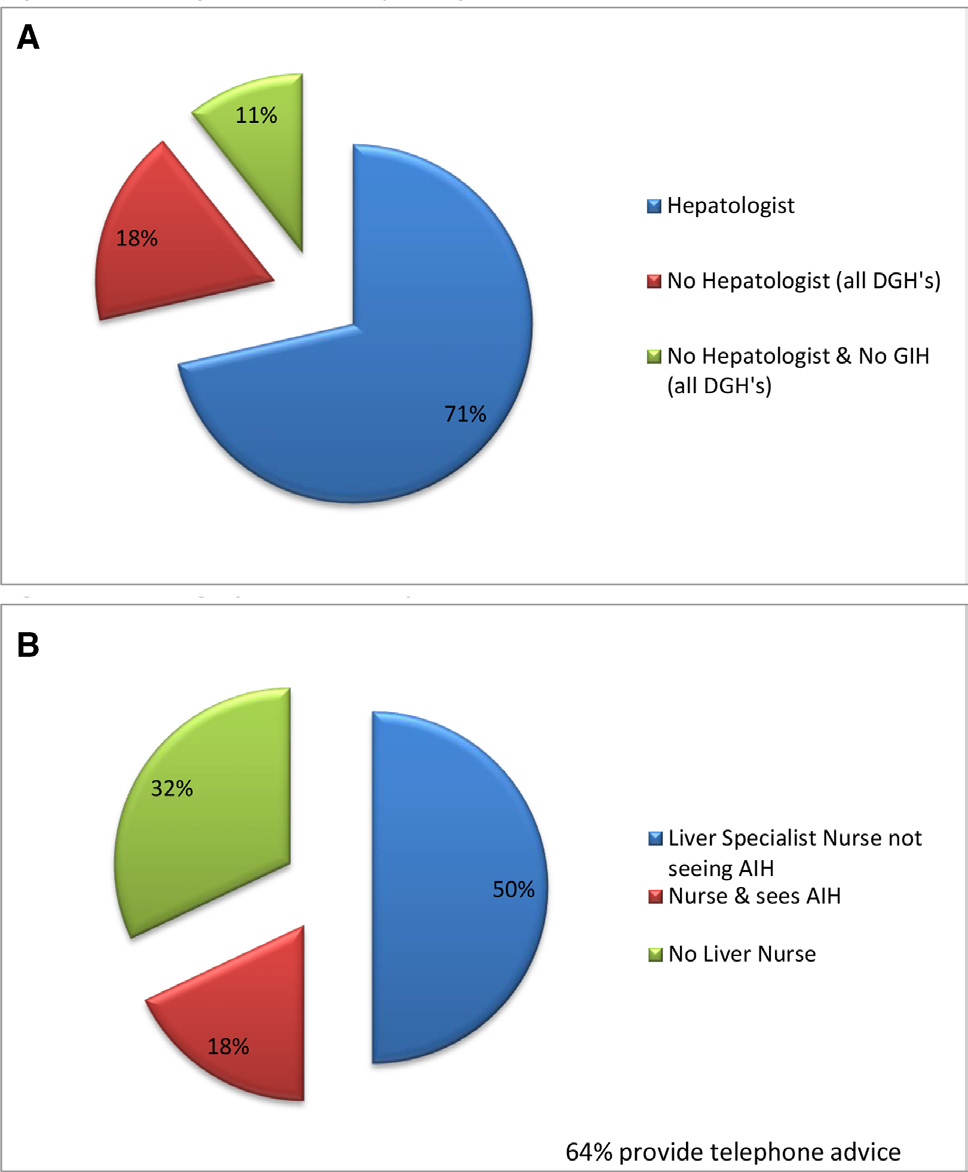
(A) Staffing: number of hepatologists. (B) Staffing: specialist nurse provision. AIH, autoimmune hepatis; DGH, district general hospital.

Eight hospitals (29%), all DGHs, had no hepatologist and three (11%) had neither a hepatologist nor a GIH (figure 1A) but were staffed by general gastroenterologists. There were 3 (0–10) consultant gastroenterologists managing patients with AIH per hospital with 30% who were GIH, and 2 (0–10) hepatologists were managing AIH. Overall, 18% (n=234) of patients were being managed in a hospital without a hepatologist.

Management of AIH was shared across 50% (18–100) of all consultants in each department. This was higher in DGHs than in UHs: 92% (20–100) versus 46%(17–100); p=0.051. In eight (29%) hospitals, management of AIH was by all the gastroenterologists and hepatologists. 7/13 (54%) hospitals, who had at least two departmental hepatologists, also had gastroenterologists seeing patients with AIH.

Nineteen hospitals had 1 (1–7) specialist nurse’s managing liver disease and 9 (32%) hospitals had no liver nurses (figure 1B). However, in only five (18%) hospitals (two DGHs) did specialist nurses see patients with AIH.

Seventeen hospitals (61%) had at least one histopathologist with an interest in liver disease. These centres were more likely to report rosettes on liver biopsy (172/795 (22%) vs 50/368 (14%); p<0.001), though other typical histological features (interface hepatitis, plasma cell infiltration and emperipolesis) were reported in similar proportion. Twenty hospitals (71%) had a joint clinical–pathological meeting.

Thirteen hospitals (46%) provided hospital patient information sheets and four (14%) had departmental guidelines for the management of AIH. Ten (36%) had a pre-existing database of patients with AIH prior to the audit.

### Standards

#### Overall cohort

We included 1267 patient cases of AIH, with a median follow-up of 3.8 (0–15) years. A summary of performance against agreed audit standards (a–h) is shown in table 2.

**Table 2:** Performance against standards in the overall cohort

Audit standard	All cases %	Standard met	% in individual centres (median (range))	Number of centres meeting standard (%)
**Treatment**				
a) ≥90% of symptomatic patients start prednisolone* within 4 months of diagnosis	92	✓	92 (33–100)	19 (68)
b) ≥90% steroids continued ≥1 year†	75	✗	76 (33–92)	3 (11)
c) ≥80% adequate blood monitoring‡	74	✗	79 (3–100)	14 (50)
d) ≥90% attain normal serum ALT by 1 year after start of treatment§	82	✗	83 (38–100)	7 (25)
e) ≥80% clinically decompensated patients who did not improve on treatment were discussed with a transplant team	95	✓	100 (80–100)	25 (100)¶
**Follow-up**				
f) ≥60% of those re-biopsied attain histological remission	37	✗	35 (0–70)	2 (8)¶
g) ≥75% do not develop de-novo cirrhosis	93	✓	95 (71–100)	27 (96)
h) ≤21% new clinical decompensation	3	✓	3 (0–10)	27 (96)

*Or equivalent (budesonide/methylprednisolone or hydrocortisone).

†In those followed up ≥1 year.

‡Liver blood tests documented in the first year at 3, 6 and 12 months adjusted for length of follow-up.

§In those with ≥12 months follow-up after treatment started and date of first normal ALT is known.

¶25of the 28 centres had decompensated patients or performed follow-up liver biopsies.

ALT, alanine aminotransferase.

**Figure 2:**
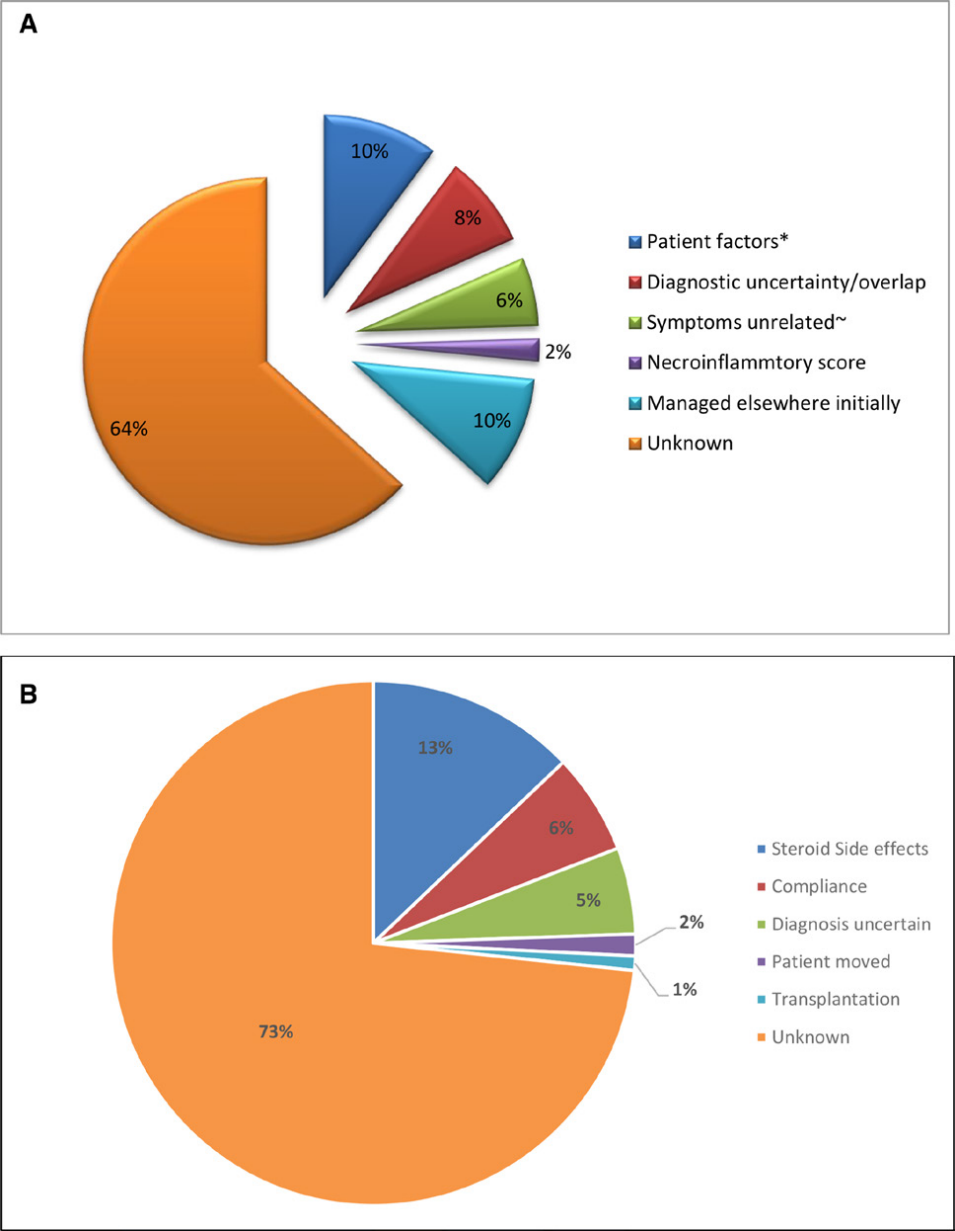
(A) Treatment standards: reasons why patients were not treated within 4 months of presenting symptoms, (B) treatment standards: reasons why prednisolone stopped before 1 year. *Patient wishes or obesity, ~clinician determined.

Time to treatment from diagnosis (defined as date of liver biopsy) in patients who were biopsied and symptomatic at presentation was 0 (0–92) months. Of 59/877 (7%) symptomatic patients were not treated with steroids, within 4 months (standard a). The reasons are shown in figure 2A.

Of patients with >12 months follow-up: 254/999 (25%) received steroids for <12 months (failing to meet Standard b). In 34 of these, steroids were stopped before alanine aminotransferase (ALT) was normalised. Reasons for stopping are given in figure 2b. Patients receiving steroids for <12 months had similar ages (52 vs 52 years) and gender distribution (83 vs 78% female; p=0.066) to those receiving steroids for longer. However, they achieve normal serum ALT more rapidly (1.9 (0-18) vs 2.4 (0–135) months; p<0.001 Mann-Whitney).

Monitoring of liver blood tests (defined as checked and recorded after 3, 6 and 12 months) on treatment was inadequate in 26% of patients (failing to meet standard c), with 50% of hospitals failing to meet the standard of ‘adequacy in 80% of patients’ and three hospitals achieving adequate monitoring in <10% of patients.

Of informative patients followed up for ≥12 months after starting treatment, 197/1066 (18%) did not achieve normal serum ALT by then (failing to meet standard d). Only 7/28 centres met the 90% standard, and in 3 (10%) centres, the percentage was less than 65%.

Standard e: ‘decompensated cases were discussed with transplant team, where appropriate’, was met in the cohort as a whole. Thus, of 150 (12%) patients who were clinically decompensated (ascites/oedema, variceal bleed or encephalopathy) either at presentation or during follow-up; 45 (30%) were discussed with or referred to a liver transplant team. Of the remaining 105 patients, 57 (38%) were aged over 70 years or had significant comorbidities and 6 (4%) were noncompliant or had excessive alcohol consumption. In 42 (28%) patients, there was no stated reason for nonreferral. Seven (5%) of these 42 patients did not improve on treatment. Two died from liver failure.

Of 333 patients undergoing follow-up liver biopsy, necroinflammatory score was ≤3 (histological remission: standard f) in 103 (37%), the percentage varied between centres: median (range) 35% (0%–70%).

Only 5% (0%–29%) of patients developed denovo during follow-up, with all but one of the centres meeting standard g. Only 3% of patients developed new clinical decompensation during follow-up, meeting standard h in all but one centre.

#### Subgroup comparisons and attainment of standards

Patients attending the 14 DGHs were (compared with those attending the 14 UHs) less likely to be treated with steroids (90 vs 94%;p=0.01) or specifically with budesonide initially (12/393 (3%) vs 49/781 (6%); p=0.01) and were more likely to experience longer delay in initiation of a steroid-sparing agent (SSA); both p=<0.05, shown in online supplemental table 1. Budesonide was the first steroid prescribed in 3% (0%–41%) of patients, with seven hospitals having no experience of using budesonide and in the centres with the highest proportions of use (≥10% of patients), all had a resident hepatologist.

10.1136/flgastro-2020-101661.supp1 Supplementary data

Patients attending UHs had lower mean serum ALT levels after 3 months (but not after 6 and 12) than those attending DGHs, despite higher starting ALT. More patients at UHs achieved at least one normal serum ALT level in the first year of follow-up; they also had greater percentage fall and percentage with normal values at 1, 3, 6 and 12 months (online supplemental table 1).

Notwithstanding these different early responses, frequency of denovo cirrhosis, relapse rate, number of relapses/year during follow-up, all cause nor liver-related death/transplant rates were significantly different between patients attending UHs and those attending DGHs either on Kaplan-Meier or Cox regression analysis (online supplemental figure 1). There were no overall differences between these different types of hospitals regarding number of standards met.

Patients attending a hospital with a specialist liver nurse received steroids for longer (82% vs 72%; p=0.001) (online supplemental table 3, standard b). Patients attending hospitals without a hepatologist were less likely to have adequate blood monitoring (69% vs 75%; p=0.04; online supplemental table 4).

## Discussion

This is the first large multicentre audit of resources and of adherence to predefined standards regarding management of AIH. Its strengths lie with the large number of participating centres of varying size and resources and, thus, may more accurately represent management of patients across the UK. The weaknesses are that many of the standards are derived from consensus opinion and failing to achieve these may not be linked with measurable poorer outcomes such as death and transplantation.

We found only limited development of subspecialisation among staffing regarding management of AIH. Nearly one-third of centres did not employ a hepatologist. In half of centres (all DGHs), at least half of the consultants managed AIH, and in 29%, all of the gastroenterologists/hepatologists did. One-third of centres have no specialist liver nurse and less than 20% have a nurse reviewing patients with AIH. The deficiency of specialist nurses for AIH contrasts that for chronic viral hepatitis and may reflect lack of funding, which for Hepatitis C has sometimes been sourced from pharma, to facilitate treatment. Unsurprisingly, UHs were better resourced with staff than DGHs. Where liver specialist nurses managed AIH, there was better adherence to corticosteroid duration treatment standards.

Many hospitals did not have a histopathologist with a specialist interest in liver disease and these centres were less likely to describe rosettes in liver biopsy reports. We previously reported^13^ probable under-reporting of rosettes in AIH, which could lead to underdiagnosis.

Of the eight predefined management standards, four (table 2, standards a, e, g and h) were met in the overall cohort; however, one (a) was met in only 68% of centres. In the symptomatic patients (8%) whose treatment was delayed by >4 months, quality of life was probably affected. Usually, the reason for delay was unclear.

Standard (e) is based on UK guidelines,^8^ which state that clinically decompensated patients should be discussed with a transplant centre unless there is a contraindication. Though the predefined standard of 80% was met, it is of concern that 5% with decompensation, not improving with treatment and apparently eligible, were not discussed with a transplant centre.

The other four predefined standards (table 2: b–d and f) were not met in the overall cohort. In 17% and 25% of cases, respectively, steroids were discontinued after <6 or 12 months; reasons provided included side effects, compliance and uncertain diagnosis, but in over 70%, the reason was unclear, although swifter normalisation of serum ALT may have influenced the shorter duration of steroids.

Continuing steroid therapy for more than 1 year has been based on fact that histological remission lags behind biochemical remission and is achieved by only half of patients after 1 year.^8^ Histological activity despite biochemical remission is associated with reduced long-term survival.^15^ However, it remains unproven that longer duration of corticosteroid therapy is associated with improved longer term outcome.^4^

Guidelines advise that patients on thiopurines and other immunosuppressive drugs should have blood monitoring at least 3 monthly,^8^ because of potential haematological, renal and hepatic impairment, especially in those with pre-existing dysfunction, and elderly patients. Half of centres fell short of this; with a wide variation in monitoring practice shown by the range of percentages (3%–100%) with adequate liver test monitoring. We did not ascertain whether those patients not having liver tests checked had separate renal and haematological monitoring, but this seems unlikely.

The numbers of patients developing new cirrhosis or new clinical decompensation during follow-up were low, but this finding could have been influenced by the relatively short median follow-up period.

Levels of noncompliance with predefined standards were not different between DGHs and UHs, suggesting that these issues are widespread. However, though not part of our standards, patients attending UHs had higher rates of steroid treatment and of budesonide use and a shorter delay in commencing SSAs. Budesonide treatment was used more frequently at UHs, and in higher proportions in centres with a hepatologist, perhaps reflecting broader clinician experience. Also, UHs had swifter ALT responses and higher rates of ALT normalisation at 1 year. Recently UK data also suggests lower remission rates in non-transplant (compared with transplant) centres (55% vs 62%).^16^

The reason for these differences is unclear. We found no association between height of pre-treatment serum ALT (higher in UHs) and its percentage fall. Initial prednisolone dose was not different in the two types of hospital. Indeed, recent evidence suggests that initial prednisolone dose does not influence serum ALT response.^17^ However, the slower ALT response in the DGHs might have been influenced by a longer delay in starting a SSA.

Despite these differences, UHs and DGHs did not differ in regards to attainment of predefined standards or in death/transplantation rate; possibly reflecting the limited follow-up time (3.8 (0–15) years). Also, failure to meet these standards may lack predictive value for death/transplantation.

However, failure to establish predictive value for mortality should not in itself invalidate the potential use of these easily measurable parameters in AIH management. Other goals of which include improving quality of life (worse in some studies where ALT normalisation is not achieved)^18^ and minimisation of steroid-related cosmetic side effects.

In conclusion, we show wide variability in service provision for AIH across UK hospitals, with more specialist physicians, histopathologists and nurses at UHs than DGHs. Often, AIH is managed by more physicians than seems necessary and there is a case for having patients with AIH under the care of a limited number of designated physicians (gastroenterologists or hepatologists).

Furthermore, several of our predefined management standards were not met either in the overall cohort or in most of the individual centres. While the importance of some in regards to patient outcome could be debated, the >4 month delay in starting treatment (although in only 8%), prevalent failure to meet blood monitoring standards and nondiscussion of eligible patients with transplant teams are of concern.

Based on our findings, there seems no compelling reason why many patients with AIH should not be managed in smaller hospitals. Though not directly supported by the data, a stronger case could be made for participation in regional networks, similar to networks for the management of patients with HCV and PBC across the UK. For AIH, this could include monthly histopathology review (via teleconference); encouraging broader discussion of clinical cases, adherence to agreed guidelines, referral of patients with less responsive disease. We also suggest that the assessment and management of patients with AIH be limited to 2–3 designated consultants with an interest in hepatology.

Finally, we suggest ongoing audit, based on developing and maintaining an outcome database. Aside from improving patient care, this will also assist hospitals in developing business cases for more resources.

## Data Availability

All data relevant to the study are included in the article or uploaded as supplementary information. All data relevant to the study are included in the article or uploaded as supplementary information. Data access for research purposes must be requested from Dr Vikki Gordon: victoria.gordon@uhcw.nhs.uk.
