# Supplementary material for: Provision and standards of care for treatment and follow-up of patients with Autoimmune Hepatitis (AIH)
Source: Frontline Gastroenterol. 2021 May 18;13(2):126–32. doi: 10.1136/flgastro-2020-101661 (PMC8862490; doi:10.1136/flgastro-2020-101661)

Supplementary Table 1: Comparison of patients attending District General Hospitals (DGH's) compared to those attending University Hospitals (UH's)*

Parameter	DGH's n=437	UH's n=830	p value
Delay between first abnormal LFTs to diagnosis (mo)±SD	9.5±17	8.6±17	ns
Delay diagnosis to start of treatment (mo)±SD	1.6±7	1.6±8	ns
Delay in starting SSA (mo)±SD	0.41±0.9	0.27±0.68	0.006
Received steroids: N (%)	393/437 (90)	779/830 (94)	0.011
Initial dose of Prednisolone (mg/day) ±SD	32.1±10	31.7±13	ns
Initial steroid Budesonide: N (%)	11/391 (3)	47/778 (6)	0.016
Mean serum ALT (IU/L) ±SD at start of treatment	336±477	461±496	<0.001
And after 28 days	113±184	100±139	ns
3 months	100±181	65±104	0.004
6 months	62±134	55±123	ns
12 months	55±252	46±89	ns
Serum ALT % change at 28 days (n)	-20±146	-63±47	<0.0001
3 months (n)	-35±108	-65±72	<0.001
6 months (n)	-37±169	-71±45	0.002
12 months (n)	-23±500	-74±30	0.02
% with normal ALT at 28 days (n)	20	62	0.007
3 months (n)	5	68	0.001
6 months (n)	36	70	0.002
12 months (n)	55	63	0.03
% with at least one normal ALT within 12 months	67	74	0.047

*Essentially identical results were obtained when patients not receiving steroids were excluded from analysis

BMJ Publishing Group Limited (BMJ) disclaims all liability and responsibility arising from any reliance placed on this supplemental material which has been supplied by the author(s)

Supplementary Table 2: Standards in University hospitals (n=830 cases) versus District General Hospitals (n=437 cases)

Standard	UH's n (%)	Standard met	DGH's n (%)	Standard met	p value
a) ≥90% of symptomatic patients start prednisolone~ within 4 months of diagnosis	540/593 (95)	✓	258/284 (91)	✓	0.9
b) ≥90% Steroids continued ≥1 year*	495/658 (75)	✗	250/341 (73)	✗	0.51
c) ≥80% adequate blood monitoring++	560/744 (75)	✗	280/389 (72)	✗	0.23
d) ≥90% attain normal serum ALT at 1 year treatment**	598/716 (84)	✗	281/360 (78)	✗	0.02
e) ≥80% clinically decompensated patients¥ who did not improve on treatment were discussed with a transplant team	103/108 (95)	✓	40/42 (95)	✓	0.97
f) ≥60% of those re-biopsied attain histological remission***	69/195 (35)	✗	34/74 (46)	✗	0.11
g) ≥75% do not develop de-novo cirrhosis	571/617 (93)	✓	308/334 (92)	✓	0.89
h) ≤21% new clinical decompensation	32/754 (4)	✓	10/405 (2.5)	✓	0.12

~or equivalent (Budesonide/methylprednisolone/hydrocortisone) as proportion of symptomatic patients. *In those followed up ≥1 year. +Liver Blood tests documented at 3, 6 & 12 months adjusted for length of follow-up. **In those with ≥12months follow up after treatment started & date of first normal ALT is known. ***Necroinflammatory score available in 195 (of 247) UH and 74 (of 86) DGH of follow-up biopsies respectively.

Supplementary Table 3: Centres with specialist nurses seeing patients (n=5) versus those attending centres without (n=23).

	Sp Nurse sees pts n=400 n (%)	Standard met	No Sp nurse seeing pts n=1148 n (%)	Standard met	p value
a) ≥90% of symptomatic patients start prednisolone~ within 4 months of diagnosis	275/291 (95)	✓	543/586 (93)	✓	0.3
b) ≥90% Steroids continued ≥1 year*	239/293 (82)	✗	506/706 (72)	✗	0.001
c) ≥80% adequate blood monitoring++	239/347 (69)	✗	601/786 (76)	✗	0.007
d) ≥90% attain normal serum ALT at 1 year treatment***	270/331 (82)	✗	609/745 (82)	✗	0.94
e) ≥80% clinically decompensated patients¥ who did not improve on treatment were discussed with a transplant team	45/48 (94)	✓	96/102 (94)	✓	0.93
f) ≥60% of those re-biopsied attain histological remission	49/123 (40)	✗	54/148 (36)	✗	0.6
g) ≥75% do not develop de-novo cirrhosis	279/303 (92)	✓	881/929 (95)	✓	0.08
h) ≤21% new clinical decompensation	14/366 (4)	✓	28/1074 (3)	✓	0.23

~or equivalent (Budesonide/methylprednisolone/hydrocortisone) as proportion of symptomatic patients.

*In those followed up ≥1 year.

++Liver Blood tests documented at 3 months, 6 months & 12 months adjusted for length of follow-up.

***In those with ≥12months follow up after treatment started & date of first normal ALT is known.

Supplementary Table 4: Centres with a Hepatologist (n=20) versus those without a Hepatologist (n=8).

	Hepatologist n=1033 patients n (%)	Standard met	No Hepatologist n=234 patients n (%)	Standard met	p value
a) ≥90% of symptomatic patients start prednisolone~ within 4 months of diagnosis	675/722 (93)	✓	143/155 (92)	✓	0.58
b) ≥90% Steroids continued ≥1 year*	612/819 (75)	✗	133/180 (74)	✗	0.81
c) ≥80% adequate blood monitoring++	694/920 (75)	✗	146/213 (69)	✗	0.04
d) ≥90% attain normal serum ALT at 1 year treatment***	744/899 (83)	✗	135/177 (76)	✗	0.04
e) ≥80% clinically decompensated patients¥ who did not improve on treatment were discussed with a transplant team	122/130 (94)	✓	19/20 (95)	✓	0.84
f) ≥60% of those re-biopsied attain histological remission	78/230 (34)	✗	25/53 (47)	✗	0.07
g) ≥75% do not develop de-novo cirrhosis	701/763 (92)	✓	178/188 (95)	✓	0.19
h) ≤21% new clinical decompensation	35/938 (4)	✓	7/221 (3)	✓	0.69

~or equivalent (Budesonide/methylprednisolone/hydrocortisone) as proportion of symptomatic patients.

*In those followed up ≥1 year.

++Liver Blood tests documented at 3 months, 6 months & 12 months adjusted for length of follow-up.

***In those with ≥12months follow up after treatment started & date of first normal ALT is known.

Supplementary Figure 1: All cause death/transplant rate in patients attending DGH's compared to UH's

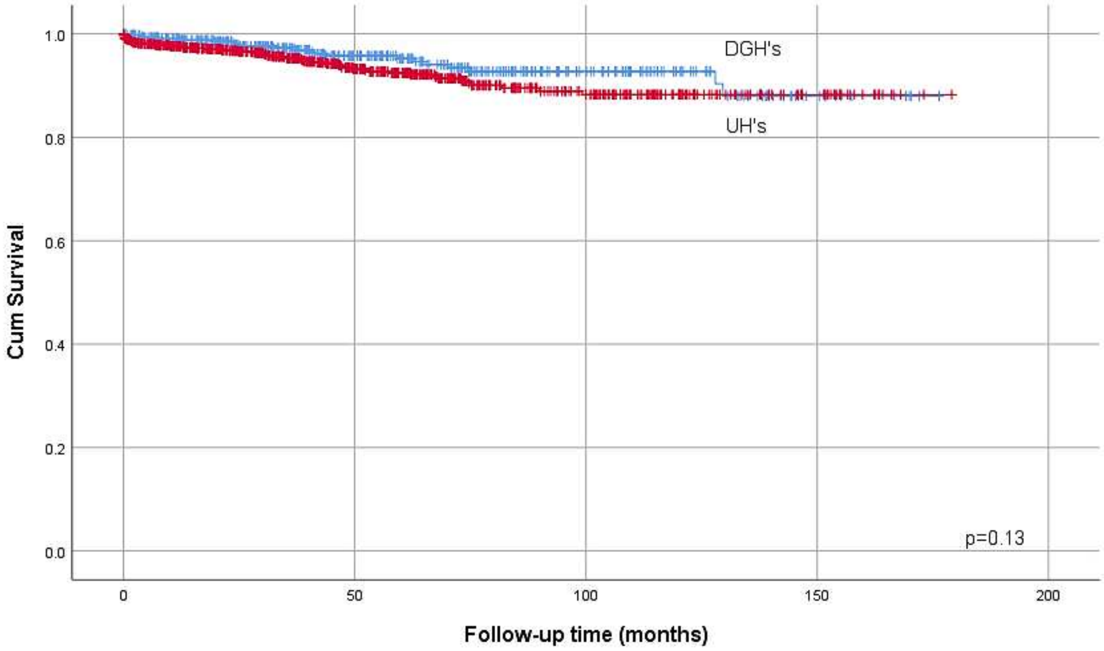

Supplement: Supplementary data [file flgastro-2020-101661supp001.pdf]
